# Comparison of Trials Using Ivermectin for COVID-19 Between Regions With High and Low Prevalence of Strongyloidiasis: A Meta-analysis

**DOI:** 10.1001/jamanetworkopen.2022.3079

**Published:** 2022-03-21

**Authors:** Avi Bitterman, Caitlin Pestana Martins, Ahuva Cices, Makarand Prasad Nadendla

**Affiliations:** 1 Department of Dermatology, Icahn School of Medicine at Mount Sinai, New York, New York; 2 Albert Einstein College of Medicine, Bronx, New York; 3 University of Denver, Denver, Colorado

## Abstract

**Importance:**

A widely cited meta-analysis of randomized clinical trials has claimed ivermectin as an effective treatment for prevention of mortality in COVID-19. However, an unrecognized interaction variable with the relative risk (RR) of mortality may substantially change the appropriate interpretation of this analysis.

**Objective:**

To evaluate the association between regional prevalence of strongyloidiasis and ivermectin trial results for the outcome of mortality by testing the hypothesis that strongyloidiasis prevalence interacts with the RR of mortality.

**Data Sources:**

Original meta-analysis as well as a manual review of all references in a dedicated ivermectin trial database (c19ivermectin) from January 1, 2019, to November 6, 2021.

**Study Selection:**

Randomized clinical trials using ivermectin as a treatment for COVID-19 and reporting the outcome of mortality. Studies were excluded in the event of publications revealing suspected trial fraud and/or randomization failure.

**Data Extraction and Synthesis:**

Study characteristics and RR estimates were extracted from each source. Estimates were pooled using random-effects meta-analysis. Differences by strongyloidiasis prevalence were estimated using subgroup meta-analysis and meta-regression. The Preferred Reporting Items for Systematic Reviews and Meta-analyses (PRISMA) guideline was followed.

**Main Outcomes and Measures:**

Relative risk of mortality in ivermectin trials in regions of high vs low strongyloidiasis prevalence and correlation coefficient of meta-regression analysis between RR of mortality and regional prevalence of strongyloidiasis.

**Results:**

A total of 12 trials comprising 3901 patients were included in the analysis. Four trials (33%) took place in regions of high strongyloidiasis prevalence and 8 (67%) trials took place in regions of low strongyloidiasis prevalence. Ivermectin trials that took place in areas of low regional strongyloidiasis prevalence were not associated with a statistically significant decreased risk of mortality (RR, 0.84 [95% CI, 0.60-1.18]; *P* = .31). By contrast, ivermectin trials that took place in areas of high regional strongyloidiasis prevalence were associated with a significantly decreased risk of mortality (RR, 0.25 [95% CI, 0.09-0.70]; *P* = .008). Testing for subgroup differences revealed a significant difference between the results of groups with low and high strongyloidiasis prevalence (χ^2^_1_ = 4.79; *P* = .03). The estimate for τ^2^ (the variance of the study effect sizes) was 0 (95% CI, 0.0000-0.2786), and the estimate for *I*^2^ (percentage of variability that is explained by between-study heterogeneity) was 0 (95% CI, 0-43.7%). The meta-regression analysis revealed an RR decrease of 38.83% (95% CI, 0.87%-62.25%) for each 5% increase in strongyloidiasis prevalence.

**Conclusions and Relevance:**

In this meta-analysis of 12 trials including 3901 patients, strongyloidiasis prevalence was found to interact with the RR of mortality for ivermectin as a treatment for COVID-19. No evidence was found to suggest ivermectin has any role in preventing mortality among patients with COVID-19 in regions where strongyloidiasis was not endemic.

## Key Points

**Question:**

Does prevalence of strongyloidiasis interact with the relative risk (RR) of mortality in ivermectin trials for the treatment of COVID-19?

**Findings:**

In this meta-analysis of 12 randomized clinical trials involving 3901 patients, favorable mortality results were limited to trials in high-prevalence regions, with no evidence that ivermectin had a mortality benefit in low-prevalence regions. Meta-regression found an association between the regional prevalence of strongyloidiasis and risk of mortality, with a decrease in RR of 39% for each 5% increase in strongyloidiasis prevalence.

**Meaning:**

Evidence supports that strongyloidiasis prevalence interacts with the RR of mortality in ivermectin trial results; no evidence was found to suggest ivermectin has any role in preventing mortality in patients with COVID-19 in regions where strongyloidiasis is not endemic.

## Teaser

This meta-analysis assesses the association between regional prevalence of strongyloidiasis and the finding of reduced COVID-19 mortality in ivermectin trials.

## Introduction

*Strongyloides stercoralis* is an intestinal helminth endemic in Latin America,^1^ Southeast Asia, and sub-Saharan Africa.^2^ *Strongyloides* hyperinfection syndrome (SHS) is a severe manifestation that occurs when autoinfection accelerates, leading to increased numbers of the parasite in the tissues involved in the autoinfection cycle.^3^ The global mean prevalence of strongyloidiasis is estimated to be 8.1%, and prevalence is highly variable across different countries.^4^ Disseminated disease occurs when the parasite spreads to organs other than those involved in its life cycle.^3,5^ The wide range of presentations combined with lack of familiarity result in SHS and disseminated disease often being misdiagnosed,^6^ and therefore the prevalence of strongyloidiasis is currently unknown.

Although SHS can occur in immunocompetent hosts,^7,8,9^ it is associated with immunosuppression, particularly from corticosteroid use. Iatrogenic corticosteroid use is commonly noted in disseminated strongyloidiasis, with a disease onset as early as 5 days and a mortality rate as high as 90%.^10^ *Strongyloides* hyperinfection syndrome has been observed after initiation of corticosteroid therapy for COVID-19.^11,12^ Of note, corticosteroids do not need to be given for disseminated strongyloidiasis to occur. For example, eosinopenia is associated with COVID-19 infections, even in patients not receiving corticosteroids,^13^ and eosinopenia is associated with risk of poor prognosis from SHS.^14^ Various recommendations have suggested that clinicians empirically treat patients with COVID-19 from strongyloidiasis*-*endemic regions with ivermectin before initiating corticosteroid therapy to prevent hyperinfection.^15^

*Strongyloides* hyperinfection syndrome is a potentially concerning interaction in ivermectin trials for the treatment of COVID-19 because these trials overwhelmingly take place in strongyloidiasis-endemic regions, and corticosteroids are often given as part of the standard care to which patients in control groups are assigned. Under ideal circumstances, all these patients would be empirically treated with ivermectin before receiving corticosteroids; however, because these patients are control patients in an ivermectin trial, this concomitant medication is prohibited. This effectively creates a study design that systematically places the control group at an increased risk of mortality compared with the treatment group, artificially causing the mortality results of the ivermectin treatment group to look favorable for the treatment of COVID-19. First, any parasites present in the treatment group are effectively treated while the untreated patients remain in the control group. Second, administration of corticosteroids as standard of care without ivermectin further amplifies the risk of hyperinfection in the control group. Third, COVID-19 itself is associated with eosinopenia even in the absence of corticosteroid use.^13^ Eosinophils play an important role in modulating parasitic infections, and eosinopenia has been associated with an increased risk of poor prognosis from SHS.^14^ Therefore, even if trials did not give corticosteroids to patients in the control group, conducting trial designs of this nature in endemic regions may still interact with outcomes. Despite taking place in strongyloidiasis-endemic regions, ivermectin trials overwhelmingly have no mention of helminthic diagnostic tests or any mention of alternative antihelminthic treatments in the control group to account for this interaction. Therefore, results of ivermectin trials conducted in strongyloidiasis-endemic regions cannot be extrapolated to patients who are not at increased risk for *Strongyloides* species infection.

## Methods

**Figure 1.**
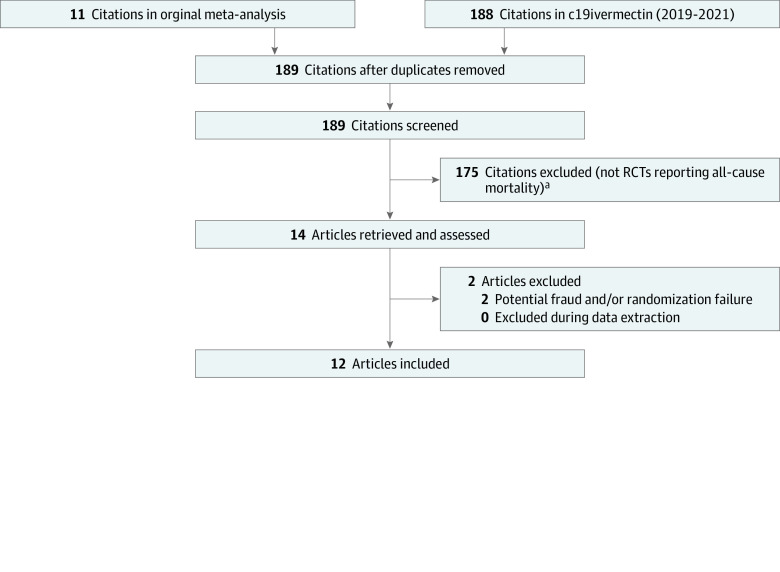
Study Flow Diagram RCT indicates randomized clinical trial. ^a^At least 1 death in either the treatment group or the control group.

This meta-analysis followed the Preferred Reporting Items for Systematic Reviews and Meta-analyses (PRISMA) reporting guideline. Based on a previously published meta-analysis,^16^ an updated subgroup analysis by regional strongyloidiasis prevalence (above and below global mean prevalence) for the primary outcome of mortality was performed. For the variable of strongyloidiasis prevalence, country-level prevalence by parasitological methods and more granular within-country regional prevalence estimates where possible were used. Data sources included the original meta-analysis as well as a manual review performed from September to November 2021, exhausting all references in a dedicated ivermectin trial database (c19ivermectin) from January 1, 2019, to November 6, 2021. Details of the search strategy and vetting process for database logging that c19ivermectin uses is detailed in eMethods in the Supplement. This manual database review was performed by 2 investigators independently (A.B. and C.P.M.). Trial characteristics and outcomes were also extracted in duplicate by 2 investigators (A.B and C.P.M.). One investigator (A.B.) assessed risk of bias of each trial. The previous meta-analysis was updated by including the results of the 3 trials^17,18,19^ released since its publication that reported mortality end points. Trials that have since come under scrutiny for trial fraud and/or randomization failure were excluded^20^ (Figure 1).

We performed Mantel-Haenszel random-effects subgroup analysis meta-analytic summation with 0.5 imputation as continuity correction for the outcome of relative risk (RR) of mortality and a mixed-effects meta-regression. Both models were performed in R, version 4.1.2,^21^ using the meta (version 5.1.1)^22^ and metafor (version 3.0.2)^23^ packages with tidyverse (version 1.3.1)^24^ for data preparation. For both the models, the data for each study’s control and intervention groups were used, including each group’s mortality events and total participants. The data were then visually inspected using box plots and scatterplots from the R package ggplot2 (version 3.3.5)^25^ to ensure that data collection was properly and accurately performed.

The random-effects subgroup analysis was performed using the studies with regional strongyloidiasis prevalence greater than or equal to the global mean (≥8.1%) in one subgroup, and below the global mean in the other subgroup (<8.1%). The model was configured with a restricted maximum likelihood estimator to estimate the τ^2^ parameter, a Q-profile method to estimate the 95% CIs for τ^2^ and τ, and a continuity correction of 0.5 in studies with zero cell frequencies. The model’s estimations for τ^2^ and *I*^2^ and a test for heterogeneity were used to assess the heterogeneity of the studies included. The τ^2^ and τ values represent the variance and standard deviation of the distribution from which the study effect sizes are drawn, respectively. The Q-profile method is a method for the estimation of the 95% CI for τ, whereas *I*^2^ represents the percentage of variability of the estimates that is accounted for by between-study heterogeneity.^26^ Finally, a test for subgroup differences was used to assess whether the subgroups’ pooled estimates for the risk ratio were statistically significantly different.

Mixed-effects meta-regression was performed, regressing the natural log RR for all-cause mortality on the regional *Strongyloides* prevalence (reported as a percentage). The model was specified with a restricted maximum likelihood estimator to estimate the τ^2^ parameter, a Q-profile method to estimate the 95% CIs for τ^2^ and τ, and a continuity correction of 0.5 in studies with zero cell frequencies. The model’s estimations for τ^2^ and *I*^2^ were used to assess the heterogeneity of the studies included. Similar to the random-effects subgroup analysis, τ^2^ and τ represent the variance and standard deviation of the distribution from which the study effect sizes are drawn, respectively. The Q-profile method is a method for the estimation of the 95% CI for τ, whereas *I*^2^ represents the percentage of variability of the estimates that is accounted for by between-study heterogeneity.^26^

Three sensitivity analyses were performed in addition to our main analysis. Two of the sensitivity analyses, which helped establish whether the model estimates and inferences were robust to the uncertainty in τ, included the use of the Knapp-Hartung estimator for the 95% CIs of the model coefficients (used when the number of studies is small to help account for the uncertainty in the estimate of τ). A permutation test on the meta-regression with 10 000 iterations was also performed to assess the robustness of model estimates and inferences in resampled data. The last test was used to assess whether our estimates and inferences were robust to inclusion only of trials using random number generators for their randomization protocols. The details of these sensitivity analyses can be found in eMethods in the Supplement.

Risk of publication bias was assessed using funnel plot analysis with the Harbord test, given the reported RR measures and the binary nature of the data.^27^ A risk-of-bias summary was performed using the Cochrane randomized clinical trial risk-of-bias tool.^28^ In addition, a test for residual heterogeneity was used to assess how much remaining heterogeneity there was after accounting for the predictor variable.

## Results

**Figure 2.**
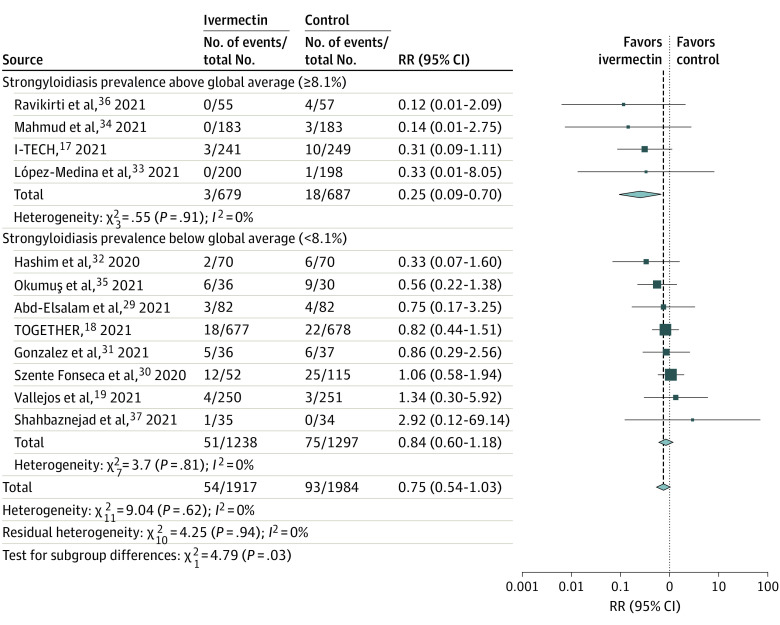
Meta-analysis of Ivermectin Trials I-TECH indicates Ivermectin Treatment Efficacy in COVID-19 High Risk Patients; RR, relative risk; TOGETHER, Early Treatment of COVID-19 With Repurposed Therapies: The TOGETHER Adaptive Platform Trial.

Twelve trials^17,18,19,29,30,31,32,33,34,35,36,37^ comprising 3901 patients were included in this analysis (Table 1). Ivermectin trials performed in areas of low regional strongyloidiasis prevalence^18,19,29,30,31,32,35,37^ were not associated with a statistically significant decreased risk of mortality (RR, 0.84 [95% CI, 0.60-1.18]; *P* = .31). By contrast, ivermectin trials that took place in areas of high regional strongyloidiasis prevalence^17,33,34,36^ were associated with a significant decreased risk of mortality (RR, 0.25 [95% CI, 0.09-0.70]; *P* = .008). Testing for subgroup differences revealed a significant difference between the results of groups with low and high strongyloidiasis prevalence (χ^2^_1_ = 4.79; *P* = .03) (Figure 2). The estimate for τ^2^ (the variance of the study effect sizes) was 0 (95% CI, 0.000-0.9432), and the estimate for *I*^2^ (percentage of variability that is explained by between-study heterogeneity) was 0 (95% CI, 0-58.3%).

**Table 1.** Characteristics of Included Studies

Source (country or region)	Design	Sample size	Ivermectin dose	Comparator	Origin of data	Strongyloidiasis prevalence, %	Corticosteroid use
Abd-Elsalam et al,^29^ 2021 (Egypt)	RCT	164	12 mg/d for 3 d	SOC	Published in PR journal	4.9^4^	As indicated per Egyptian Ministry of Health SOC
Szente Fonseca et al,^30^ 2020 (North Brazil)	Double-blind	167	14 mg/d for 3 d (plus placebo for 2 d)	Hydroxychloroquine, 400 mg BID, on day 0 then daily for 4 d; chloroquine, 450 mg BID on day 0 then daily for 4 d	Published in PR journal	5.3^1^	97% in experimental group, 98%-100% in control group
Gonzalez et al,^31^ 2021 (Mexico)	Double-blind	73	12 mg once	Placebo	*medRxiv* preprint	7.0^4^	58.3% in experimental group, 51.3% in control group
Hashim et al,^32^ 2020 (Iran)	Quasi-RCT	140	0.2 mg/kg for 2 d with or without third dose 1 wk later	SOC	Published in PR journal	5.3^4^	Dexamethasone, 6 mg/d, or methylprednisolone, 40 mg BID, if indicated
I-TECH,^17^ 2021 (Malaysia)	RCT	490	0.4 mg/kg daily for 5 d	SOC	Preliminary report by Ministry of Health of Malaysia	15.9^4^	26.9% in experimental group, 26.5% in control group
López-Medina et al,^33^ 2021 (Colombia)	Double-blind	398	0.3 mg/kg for 5 d	Placebo	Published in PR journal	18.4^4^	3% in experimental group, 6.1% in control group
Mahmud et al,^34^ 2021 (Bangladesh)	Double-blind	366	12 mg in single dose	Placebo plus SOC	Published in PR journal	17.3^4^	As indicated per local SOC guidelines
Okumuş et al,^35^ 2021 (Turkey)	RCT	66	0.2 mg/kg for 5 d	SOC	Published in PR journal	5.6^4^	Unknown
Ravikirti et al,^36^ 2021 (India)	Double-blind	112	12 mg for 2 d plus SOC	Placebo plus SOC	Published in PR journal	10.4^4^	All patients received at least 1 dose
Shahbaznejad et al,^37^ 2021 (Iran)	Double-blind	69	0.2 mg/kg for 1 dose	SOC	Published in PR journal	4.8^4^	Unknown
TOGETHER,^18^ 2021 (Southeast Brazil)	RCT	1355	400 μg/kg to 90 kg of weight daily for 3 d	Placebo	Presentation published online	3.9^1^	Unknown
Vallejos et al,^19^ 2021 (Argentina)	RCT	501	Patients weighing ≤80 kg: 12 mg/d for 2 d; patients weighing 80-110 kg: 18 mg/d for 2 d; patients weighing >110 kg: 24 mg/d for 2 d	SOC	Published in PR journal	5.1^4^	4.8% in experimental group, 4.4% in control group

Abbreviations: BID, twice daily; I-TECH, Ivermectin Treatment Efficacy in COVID-19 High-Risk Patients; PR, peer-reviewed; RCT, randomized clinical trial; SOC, standard of care; TOGETHER, Early Treatment of COVID-19 With Repurposed Therapies: The TOGETHER Adaptive Platform Trial.

**Figure 3.**
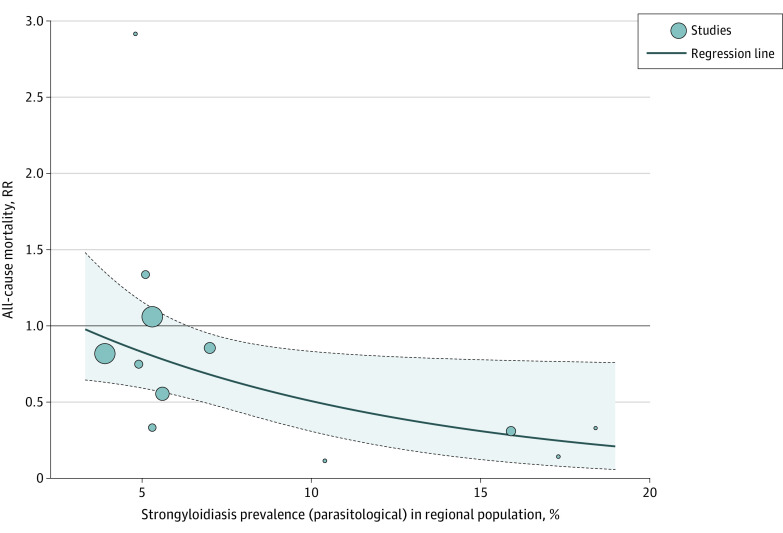
Meta-regression Analysis of Ivermectin Trials The shaded region within the dashed lines represents the 95% CIs. RR indicates relative risk.

The meta-regression analysis revealed a linear coefficient of −0.0983 (*P* = .046) for the strongyloidiasis prevalence and the natural log RR for all-cause mortality. From this, the decrease in RR for each 5% increase in strongyloidiasis prevalence was calculated to be 38.83% (95% CI, 0.87%-62.25%) (Figure 3). The estimate for τ^2^ (the variance of the study effect sizes) was 0 (95% CI, 0.0000-0.2786), and the estimate for *I*^2^ (percentage of variability that is explained by between-study heterogeneity) was 0 (95% CI, 0-43.7%). Testing for residual heterogeneity returned a test statistic of QE_10_ = 5.06 (*P* = .89). Testing for assumptions of linearity and residual distribution checks are described in eFigures 1 and 2 in the Supplement.

In addition, no qualitative differences were found in our estimates and inferences within the 3 sensitivity analyses, 2 of which (the Knapp-Hartung estimator and permutation analysis) were used to help account for uncertainty in our estimate of τ and 1 of which was used to assess the exclusion of trials not using random number generators. The results of the first 2 analyses suggested that our main model’s estimates and inferences were not qualitatively different with respect to uncertainty in the estimate of τ. The results of the third sensitivity analysis suggest that our model’s estimates and inferences did not change qualitatively, despite inclusion of trials using random number generators (eFigures 3 and 4 in the Supplement).

A risk-of-bias summary is provided in Table 2. Assessment of risk of publication bias using funnel plot analysis with the Harbord test did not show funnel plot asymmetry (*P* = .16) (eFigure 5 in the Supplement).

**Table 2.** Risk-of-Bias Summary

Source	Risk of bias by item^a^						
	Random sequence generation	Allocation concealment	Blinding of participants and personnel	Blinding of outcome assessment	Incomplete outcome data	Selective reporting	Other bias
Abd-Elsalam et al,^29^ 2021	Low	High	High	High	Low	Low	Unclear
Szente Fonseca et al,^30^ 2020	Low	Low	Low	Low	Low	Low	Low
Gonzalez et al,^31^ 2021	High	High	High	Unclear	Unclear	Unclear	High
Hashim et al,^32^ 2020	High	High	High	High	Low	Low	Unclear
I-TECH et al,^17^ 2021	Unclear	Unclear	Unclear	Unclear	Unclear	Unclear	Unclear
López-Medina et al,^33^ 2021	Low	Low	Low	Low	Low	Low	Low
Mahmud et al,^34^ 2021	Low	Low	Low	Low	Low	Low	Low
Okumuş et al,^35^ 2021	High	Unclear	High	Unclear	Low	Low	Low
Ravikirti et al,^36^ 2021	Low	Low	Low	Unclear	Unclear	Low	Low
Shahbaznejad et al,^37^ 2021	Low	Low	Low	Unclear	Unclear	Unclear	Unclear
TOGETHER,^18^ 2021	Unclear	Unclear	Unclear	Unclear	Unclear	Unclear	Unclear
Vallejos et al,^19^ 2021	Low	Low	Low	Low	Low	Low	Low

Abbreviations: I-TECH, Ivermectin Treatment Efficacy in COVID-19 High-Risk Patients; TOGETHER, Early Treatment of COVID-19 With Repurposed Therapies: The TOGETHER Adaptive Platform Trial.

^a^ Determined by reviewer judgment for each trial.

## Discussion

Consistent with the findings of the subgroup analyses and meta-regression, an association between the observed mortality benefits of ivermectin dependent on the regional prevalence of strongyloidiasis was found. This argues in favor of the hypothesis that strongyloidiasis prevalence interacts with the RR of mortality in ivermectin trials for the outcome of mortality, rather than having a treatment effect on COVID-19 per se.

In future research assessing potential mechanistic explanations for viral clearance, this interaction should also be kept in mind, given that helper T cell 2 (T_H_2) immune responses driven by helminth parasites may improve clinical outcomes at the cost of slower viral clearance, whereas treating such parasites alleviates the T_H_2 response, allowing for a more robust T_H_1 response to accelerate viral clearance at the cost of T_H_1 cytokine storm–related responses that may worsen clinical outcomes.^38^ Of course, the administration of corticosteroids in the presence of *Strongyloides* infection would be expected to supersede in terms of clinical risk. Thus, even if future trials indicate an increased viral clearance, it may be the case that ivermectin in and of itself has no inherent effect on viral clearance, resulting in another end point that will not extrapolate to nonendemic regions. Indeed, even without the use of corticosteroids, *Strongyloides* infection may still interact with the RR of mortality, because the treatment group is still receiving standard care for a given condition whereas the control group is not. This may be less likely to affect mortality without corticosteroids, but secondary outcomes may be impacted.

In line with prior recommendations, it is prudent that patients at risk for strongyloidiasis be empirically treated with ivermectin before the initiation of corticosteroid therapy.^15,39^ In the context of a trial wherein ivermectin is the treatment, there are several options to consider. One option is a trial design wherein an alternative antihelminthic other than ivermectin is used. However, this may not be ideal, because evidence suggests that alternatives such as albendazole are not as effective at treatment compared with ivermectin, and the strength of the evidence is of weaker certainty for thiabendazole efficacy.^40^ The ideal scenario to handle this interaction is to simply perform trials in nonendemic regions. Finally, institutional review boards should consider the ethical implications of trials designed with a control group resulting in substandard therapy.

### Limitations

There are several limitations to our analysis. First, the state of ivermectin trial publications at large is tenuous, with several trials coming under heavy scrutiny for egregious violations, including fraud. For this reason, a conservative approach in including studies was taken, excluding studies under scrutiny of trial fraud, and a sensitivity analysis only including trials with appropriate randomization protocols was performed. Second, details on the proportion of patients given corticosteroids (which may serve as a confounder or an interaction with the RR of mortality in its own right) in each trial were not clear for all trials, precluding the inclusion of this variable in the regression model. Third, low event counts in the trials may make the results less reliable. Fourth, varying trial recruitment across urban and rural populations (where strongyloidiasis prevalences often differ) may diminish the reliability of strongyloidiasis trial prevalence estimates. Despite these limitations, the findings warrant concern for ivermectin trials for the treatment of COVID-19 that are not designed to address this interaction.

## Conclusions

In this meta-analysis of 12 trials comprising 3901 patients, strongyloidiasis prevalence was found to interact with the RR of mortality when ivermectin was used as a treatment for COVID-19. No evidence was found to suggest that ivermectin has any role in preventing mortality in patients with COVID-19 in regions where strongyloidiasis is not endemic. Results of ivermectin trials in strongyloidiasis-endemic regions may not extrapolate to strongyloidiasis-nonendemic regions. Future trials in nonendemic regions may provide insight into the true effect of ivermectin in this context. In the interim, we strongly caution against extrapolation for patients not at increased risk for strongyloidiasis.

## Notes

Supplement. **eMethods.** Subgroup and Sensitivity Analyses and Database Search Details
eReferences
**eFigure 1.** Meta Regression Assumptions: Linearity Check **eFigure 2.** Meta Regression Assumptions: Residual Distribution Check **eFigure 3.** Sensitivity Analysis Excluding Trials With High Risk of Bias Due to Randomization **eFigure 4.** Sensitivity Analysis Excluding Trials With High Risk of Bias Due to Randomization Protocols (Sensitivity Analysis Meta-regression) **eFigure 5.** Funnel Plot Assessing Publication Bias
