# Supplementary material for: Comparison of Trials Using Ivermectin for COVID-19 Between Regions With High and Low Prevalence of Strongyloidiasis: A Meta-analysis
Source: JAMA Netw Open. 2022 Mar 21;5(3):e223079. doi: 10.1001/jamanetworkopen.2022.3079 (PMC8938718; doi:10.1001/jamanetworkopen.2022.3079)
Supplement: Supplement. — eMethods. Subgroup and Sensitivity Analyses and Database Search Details eReferences eFigure 1. Meta Regression Assumptions: Linearity Check eFigure 2. Meta Regression Assumptions: Residual Distribution Check eFigure 3. Sensitivity Analysis Excluding Trials With High Risk of Bias Due to Randomization eFigure 4. Sensitivity Analysis Excluding Trials With High Risk of Bias Due to Randomization Protocols (Sensitivity Analysis Meta-regression) eFigure 5. Funnel Plot Assessing Publication Bias [file jamanetwopen-e223079-s001.pdf]

Supplementary Online Content

Bitterman A, Martins CP, Cices A, Nadendla MP. Comparison of trials using ivermectin for COVID-19 between regions with high and low prevalence of strongyloidiasis: a meta-analysis. *JAMA Netw Open*. 2022;5(3):e223079.
doi:10.1001/jamanetworkopen.2022.3079

eMethods. Subgroup and Sensitivity Analyses and Database Search Details

eReferences

eFigure 1. Meta Regression Assumptions: Linearity Check

eFigure 2. Meta Regression Assumptions: Residual Distribution Check

eFigure 3. Sensitivity Analysis Excluding Trials With High Risk of Bias Due to Randomization

eFigure 4. Sensitivity Analysis Excluding Trials With High Risk of Bias Due to Randomization Protocols (Sensitivity Analysis Meta-regression)

eFigure 5. Funnel Plot Assessing Publication Bias

This supplementary material has been provided by the authors to give readers additional information about their work.

© 2022 Bitterman A et al. *JAMA Network Open*.

eMethods. Subgroup and Sensitivity Analyses and Database Search Details

Given the nature of the trials, their protocols, locations, and reported results, it was decided to have the model of random effects for the subgroup analysis.

The assumptions of the meta regression regressing natural log relative risk of all-cause mortality on strongyloides prevalence are assessed here. From the knowledge of the studies, it was assumed a priori that the studies had the same $\tau 2$ value, were independent from each other, and that there was little to no measurement error within the individual studies. The approximate linear relationship between the log relative risk and the strongyloides prevalence was assessed via a scatter plot. (eFigure 1)

The distribution of the $\sigma 2$ (sampling error) was assessed via the fitted values vs standardized residuals plot. (eFigure 2). The distribution of the error terms were reasonably met, as the variance is relatively equal across the fitted values.

As a sensitivity analysis, a permutation analysis (useful for assessing whether there is overfit by reassessing the model on resampled data) was performed on our main meta regression model. The permutation analysis on the model returned an adjusted p-value of 0.0136 for the linear coefficient of -0.0983 of strongyloides prevalence on the natural log relative risk, suggesting that the model is not overfit and is robust to the uncertainty for our estimate of τ.

As another sensitivity analysis, a Knapp-Hartung adjustment was considered for both the main subgroup analysis and main meta regression models. **(Knapp, Guido, and Joachim Hartung. 2003. "Improved Tests for a Random Effects Meta-Regression with a Single Covariate."** ***Statistics in Medicine*** **22 (17): 2693–2710).** This was due to the lower number of studies, the uncertainty in the estimate of τ , the difference in precisions between studies.

© 2022 Bitterman A et al. *JAMA Network Open*.

However, given the estimation of τ2 being 0 and the issues associated with the modification when τ2 is 0[1], we decided to default to the results without the modification. However, we will provide the changes in results with the modification below:

For the subgroup analysis (Knapp-Hartung), Ivermectin trials that took place in areas of low regional strongyloides prevalence were not associated with a significant decreased risk of mortality, Relative Risk (RR) = 0.8397 [0.6252; 1.1280] (P = 0.2). By contrast, ivermectin trials that took place in areas of high regional strongyloides prevalence were associated with a significant decreased risk of mortality, RR = 0.2507 [0.1225; 0.5129] (P < 0.01). Test for subgroup differences revealed a significant difference between the results of low and high strongyloides prevalence groups. $Chi^2 = 22.09$, (P < 0.0001).

In addition, the meta regression (Knapp-Hartung) analysis revealed a linear coefficient of -0.0983, (P=0.0186) for strongyloides prevalence (percent)'s effect on the natural log relative risk for all-cause mortality. The estimates and confidence intervals for τ2 and I2 were the same as the main model. The additional permutation analysis for the model with the Knapp-Hartung adjustment returned an adjusted p-value of 0.0145 for the linear coefficient of -0.0983 of strongyloides prevalence on the natural log relative risk, suggesting that the model's estimates and resulting inferences would not qualitatively change.

We decided to perform a sensitivity analyses that excluded trials with improper randomization protocols, which excluded three trials (Hashim, Gonzalez, and Okumus). The subgroup analysis and meta regression results are shown below:

For the subgroup analysis, ivermectin trials that took place in areas of low regional strongyloides prevalence were not associated with a significant decreased risk of mortality, RR = 0.96 [0.6466; 1.4254] (P = 0.84). By contrast, ivermectin trials that took place in areas of high

© 2022 Bitterman A et al. *JAMA Network Open*.

regional Strongyloides prevalence were associated with a significant decreased risk of mortality, RR = 0.25 [0.09-0.70] ($P < 0.01$). Test for subgroup differences revealed a significant difference between the results of low and high Strongyloides prevalence groups. $Chi^2 = 5.7$, ($P = 0.017$). (eFigure 3) The estimate and confidence interval for τ2 (the variance of the study effect sizes) was 0 [0.0000; 2.3662] and the estimate and confidence interval for I2 (percentage of variability that is explained by between-study heterogeneity) was 0.0% [0.0%; 64.8%]. While the τ2 and I2 are smaller than assumed to be a priori, the confidence intervals indicate that τ2 and I2 are compatible with values up to 2.3662 and 64.8%, respectively.

The meta regression in the sensitivity analysis revealed a linear coefficient of -0.1044, (P=0.0350) for the Strongyloides prevalence effect on the natural log relative risk for all-cause mortality. From this, the estimated relative risk percent decrease for each 5% increase in strongyloides prevalence was calculated to be 40.68% [3.6% - 63.49%]. (eFigure 4) The estimate and confidence interval for τ2 (the variance of the study effect sizes) was 0 [0.0000 - 0.5341], and the estimate and confidence interval for I2 (percentage of variability that is explained by between-study heterogeneity) was 0.0% [0.0% - 57.0%]. While the τ2 and I2 are smaller than assumed to be a priori, the confidence intervals indicate that τ2 and I2 are compatible with values up to 0.5341 and 57.0%, respectively. Since there was no estimated heterogeneity, the test for residual heterogeneity predictably returned a test statistic of QE(df = 10) = 2.94 (P=0.8905). The additional permutation analysis returned an adjusted p-value of 0.0132 for the linear coefficient of -0.0983 of strongyloides prevalence on the natural log relative risk, suggesting that the model estimates and resulting inferences would not qualitatively change.

The funnel plot analysis with the Harbord test did not show significant funnel plot asymmetry (p = 0.1568) (eFigure 5).

© 2022 Bitterman A et al. *JAMA Network Open*.

Database search details:

c19ivermectin is a dedicated database logging ivermectin trials for the treatment of COVID-19. These trials are procured by a number of sources including automated searches (via ivmmeta) of PubMed, medRxiv, ClinicalTrials.gov, The Cochrane Library, Google Scholar, Collabovid, Research Square, ScienceDirect, Oxford University Press, the reference lists of other studies and meta-analyses, and submissions to the site c19ivermectin.com, which regularly receives submissions of studies upon publication. Search terms include ((“ivermectin”) AND (“COVID-19” OR “SARS-CoV-2”)), OR (“ivermectin”), with inclusion of items regarding the use of ivermectin for COVID-19 that report a comparison with a control group. Investigators A.B. and C.P.M. performed a manual review between September 2021 and November 2021, exhausting all references in the dedicated Ivermectin trial database, c19ivermectin from January 1st 2019 until November 6th, 2021.

Analysis code:

The code to all the analyses can be found at https://github.com/makarandnadendla/iver_strongo_meta

eReferences

1. Jackson D, Law M, Rücker G, Schwarzer G. The Hartung-Knapp modification for random-effects meta-analysis: A useful refinement but are there any residual concerns? Statistics in Medicine 2017;**36**(25):3923-34 doi: 10.1002/sim.7411[published Online First: Epub Date]|.

© 2022 Bitterman A et al. *JAMA Network Open*.

eFigure 1. Meta Regression Assumptions: Linearity Check

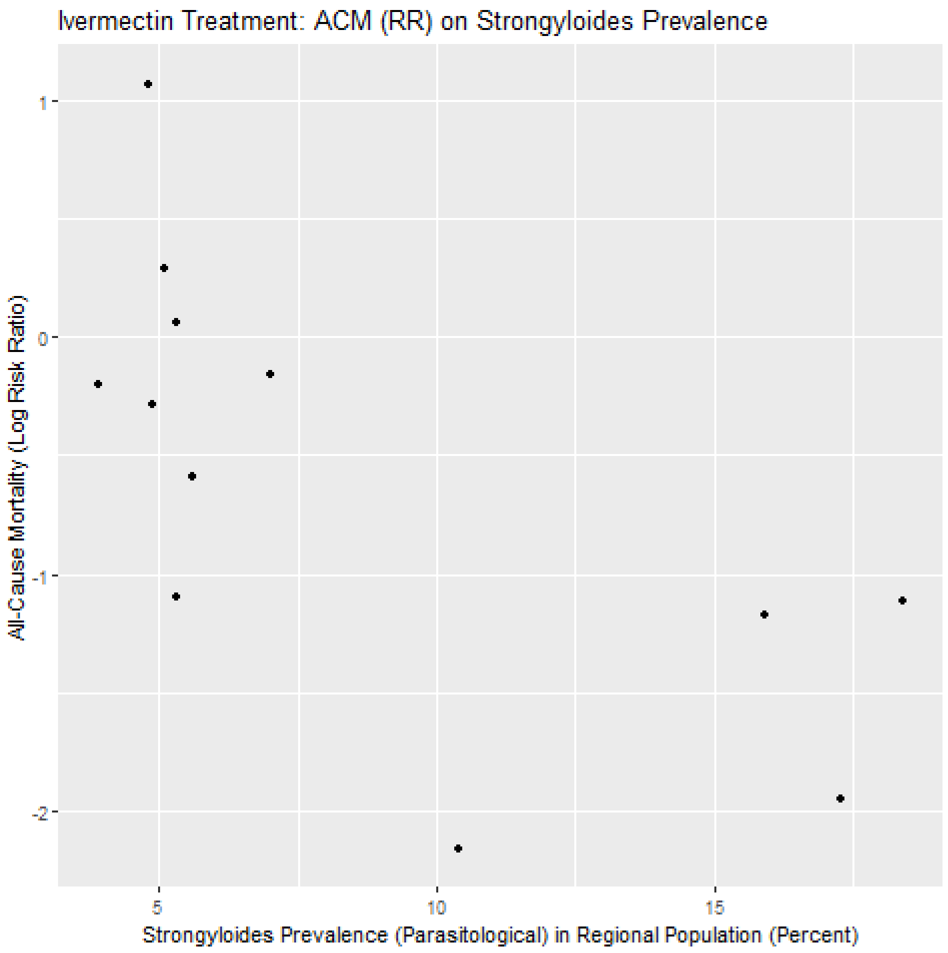

© 2022 Bitterman A et al. *JAMA Network Open*.

eFigure 2. Meta Regression Assumptions: Residual Distribution Check

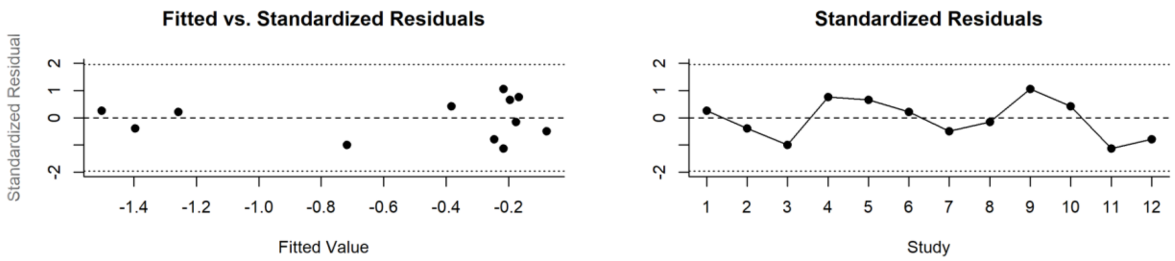

eFigure 3. Sensitivity Analysis Excluding Trials With High Risk of Bias Due to Randomization

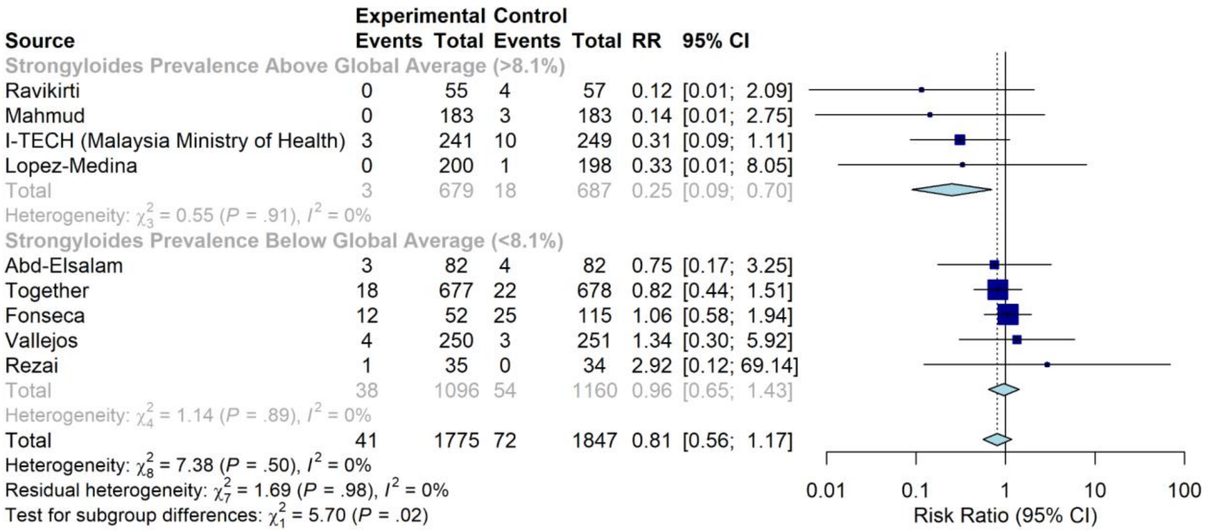

© 2022 Bitterman A et al. *JAMA Network Open*.

eFigure 4. Sensitivity Analysis Excluding Trials With High Risk of Bias Due to Randomization Protocols (Sensitivity Analysis Meta-regression)

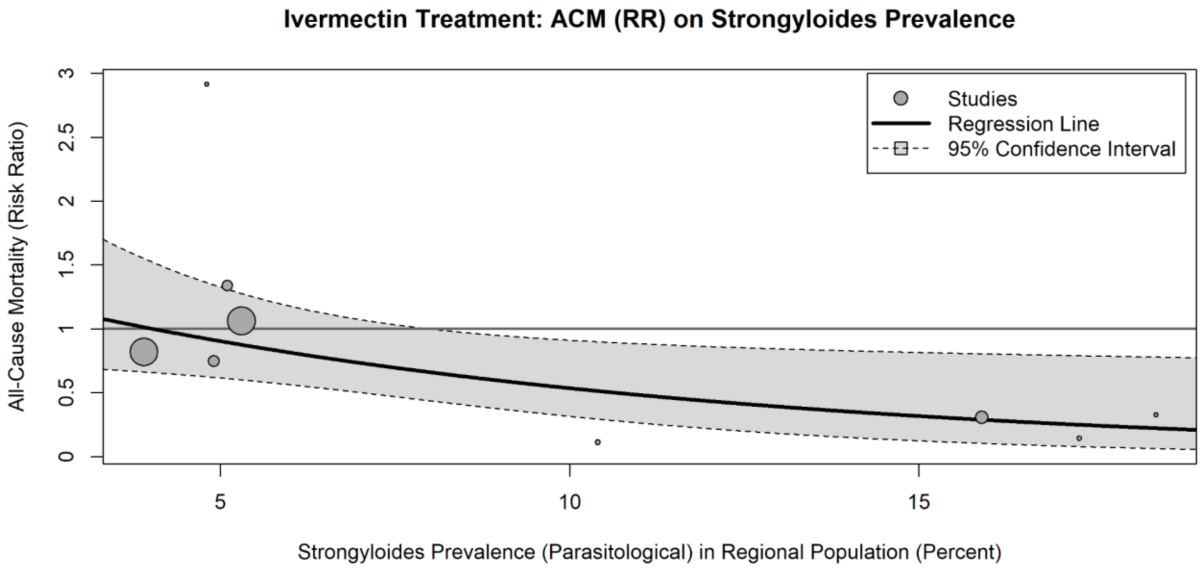

eFigure 5. Funnel Plot Assessing Publication Bias

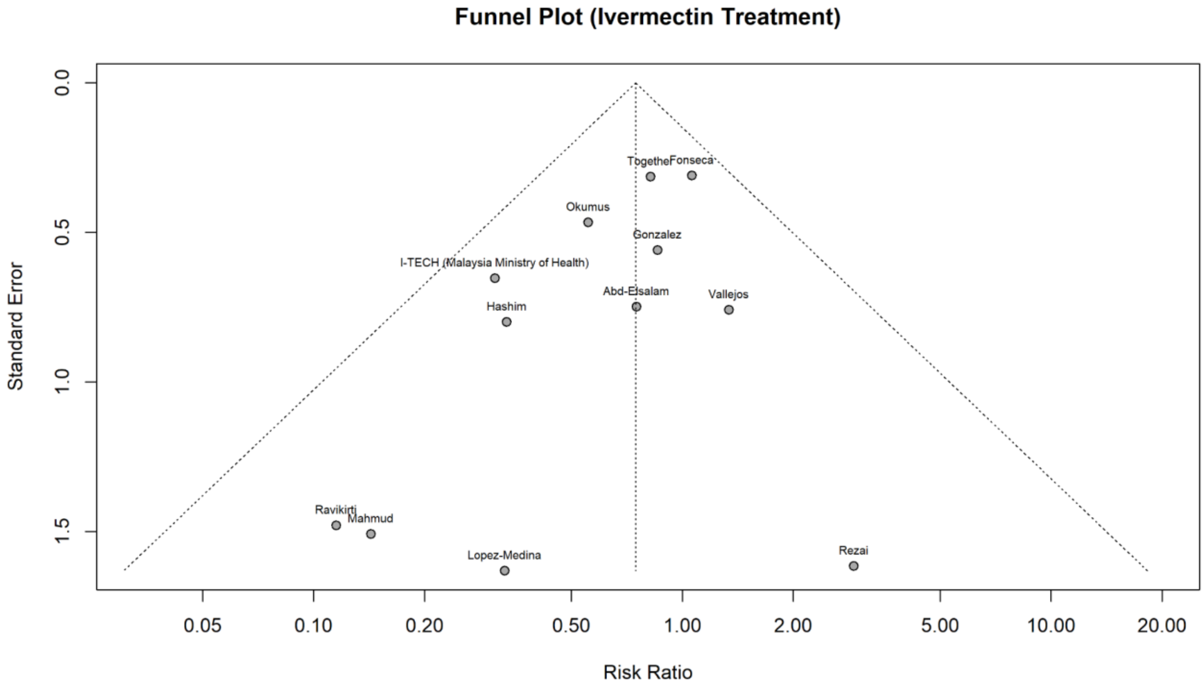

© 2022 Bitterman A et al. *JAMA Network Open*.
